# Median-to-Ulnar Nerve Communication in Carpal Tunnel Syndrome: An Electrophysiological Study

**DOI:** 10.3390/neurolint13030031

**Published:** 2021-07-12

**Authors:** Vincenzo Di Stefano, Andrea Gagliardo, Filomena Barbone, Michela Vitale, Laura Ferri, Antonino Lupica, Salvatore Iacono, Antonio Di Muzio, Filippo Brighina

**Affiliations:** 1 Department of Biomedicine, Neuroscience and Advanced Diagnostic (BIND), University of Palermo, 90127 Palermo, Italy; andrigl@gmail.com (A.G.); antlupica@gmail.com (A.L.); Salvo.iak@gmail.com (S.I.); filippobrighina@gmail.com (F.B.); 2 Department of Neuroscience, Imaging and Clinical Sciences, “G. d’Annunzio” University, 66013 Chieti, Italy; agapefilo@gmail.com (F.B.); michela.vitale1@yahoo.it (M.V.); ferrilaura@outlook.it (L.F.); 3 Department of Neurology, “SS Annunziata” Hospital, 66100 Chieti, Italy; antoniodimuzio1@gmail.com

**Keywords:** Martin-Gruber anastomosis, carpal tunnel syndrome, ulnar neuropathy at elbow, median nerve, ulnar nerve, neurophysiology

## Abstract

The median-to-ulnar communicating branch (MUC) is an asymptomatic variant of the upper limb innervation that can lead to interpretation errors in routine nerve conduction studies. The diagnosis of carpal tunnel syndrome (CTS) or ulnar nerve lesions can be complicated by the presence of MUC. In this study, we describe electrophysiological features of MUC in CTS patients presenting to our clinic. We enrolled MUB cases from consecutive CTS patients referred to our laboratory between the years 2014 and 2019. MUC was present in 53 limbs (36 patients) from the studied population. MUC was bilateral in 53% of patients. MUC type II was the most common subtype (74%), followed by types III and I; more coexisting MUC types were found in the majority of tested limbs. A positive correlation was demonstrated between the severity of CTS and the presence of positive onset, faster CV, or a double component of the compound muscle action potentials. We emphasize the importance of suspecting the presence of MUC in CTS in the presence of a positive onset or a double component in routine motor conduction studies.

## 1. Introduction

The median-to-ulnar communicating branch (MUC) [1], also known as “Martin-Gruber anastomosis/communication,” is a common anatomical innervation variant of the upper limb in which a crossover of axons passes from the median (MN) to the ulnar nerve (UN) in the forearm; this condition occurs asymptomatically in about 5–40% of the population and is bilateral in 10–40% of cases [2,3,4,5,6,7,8,9,10]. MUC usually goes undetected, but sometimes it is revealed by the unusual distribution of a motor or sensory deficit after injuries of the MN and UN [11]. The fibers involved in MUC, which are usually motor fibers [2,12,13], come from the C8 to T1 nerve roots, travel in the main trunk of the MN or the anterior interosseous nerve, and join the UN in the forearm at 3–10 cm distal to the medial epicondyle of the humerus [2,6,14,15]. According with the classification proposed by Oh, three electrophysiological MUC subtypes can be recognized depending on the distribution of the MUC axonal fibers in the hand, with specific electrophysiological features in motor conduction studies of the MN and UN for each MUC subtype [16] (Table 1). In the absence of communication, the stimulation of the MN and UN evokes a similar response at the wrist and elbow. Conversely, when MUC is present, the response from MN stimulation is smaller at the wrist compared to elbow stimulation, because many axons have already crossed from the MN to the UN [12,16]. The opposite happens with UN stimulation, as the elbow response is smaller because the UN receives crossing fibers in the forearm, resulting in a bigger response at the wrist [6,17]. Hence, MUC might be rarely misinterpreted as a conduction block of the UN between the wrist and below-elbow sites [17,18,19,20]. Of interest, MUC may lead to interpreting errors during routine nerve conduction studies (NCS) in patients with carpal tunnel syndrome (CTS) [5,11,12,21,22]. In fact, in the presence of CTS, the compound motor action potential (CMAP) at the elbow could present an initial positive deflection, thus leading to an apparently fast nerve conduction velocity (CV) of the MN in the forearm [5,8,11,21]. These alterations are not always easy to recognize despite marked discrepancies between clinical and electrodiagnostic findings. Sometimes, failure in diagnosis can result in an underrating of CTS severity or unnecessary surgical decompression in the case of UN neuropathy [11,17,20]. In this study, we describe CTS patients with neurophysiological evidence of MUC to characterize its electrophysiological features and its impact in clinical practice.

## 2. Materials and Methods

### 2.1. Aims of the Study

In this study, we describe the electrophysiological features of median-to-ulnar nerve communication in a cohort of CTS patients to better characterize MUC’s electrophysiological features and its impact in clinical practice. Furthermore, we explore the role of several grades of CTS severity on the presence of MUC and its influence on motor conduction studies parameters for each patient.

### 2.2. Participants and Data Collection

Among patients referred to our electrophysiology laboratory for the evaluation of CTS between 2014 and 2019, we only selected the patients with at least two definite electrodiagnostic criteria for MUC and the clinical diagnosis of CTS [23] (Table 2). Hence, patients with CTS satisfying the criteria for MUC underwent a complete NCS protocol for the evaluation of MUC. For each enrolled patient, we only collected data for motor nerves such as CMAP amplitude (measured from the baseline to the negative peak) [9,15,24], distal motor latency (DML) and CV, whereas sensory nerve action potential (SNAP) collection was only performed for the MN to assess CTS severity.

### 2.3. Electrophysiology Procedures

NCS were performed according to standard procedures (i.e., bipolar surface stimulating electrodes delivering rectangular pulses 0.1–0.5 ms in duration and recording electrodes placed over the recording site, with a ground electrode placed between recording and stimulation electrodes) [16,23,24]. In particular, for MUC detection, the study protocol was defined as follows: (1) stimulation of the MN at the wrist and elbow and recording from APB, ADM, and FDI muscles in three different stimulation trials; (2) stimulation of the UN at the wrist and below the elbow at least 4 cm distal to the medial epicondyle of the humerus and recording from APB, ADM, and FDI muscles in three different stimulation trials. As we studied MUC through recordings from APB, ADM, and FDI muscles, we used the classification system presented by Oh (Table 1) [16]. We evaluated several neurophysiological parameters as reliable measures of the communication entity: the presence of a positive onset, a double potential, CV, CMAP amplitude, “gain” in CMAP amplitude obtained by a distal UN stimulation compared to a proximal stimulation, and “drop” in CMAP amplitude obtained by a proximal MN stimulation compared to a distal stimulation. Finally, the severity of CTS was assessed according to consensus criteria [25]. 

### 2.4. Statistical Analyses

We reported continuous variables as mean with standard deviation (SD) and categorical variables as percentages. We compared categorical variables (presence of double potentials, positive onset, and MUC subtypes) among groups with the Chi-square test, whereas Pearson correlation coefficient was used for grade of correlations between categorical variables. The continue variables were analyzed using the ANOVA, with the between-subjects factor group (5 levels: normal, minimal, slight, moderate, and severe CTS) and the within-subjects factor group (CMAP amplitude, CV, DML, ulnar gain in amplitude, and median drop in amplitude). Statistical analyses were performed using the SPSS software (version 26.0 IBM Statistics, IBM Corp); the level of significance was set at a *p* value of <0.05.

## 3. Results

From 941 patients referred to our laboratory for CTS between 2014 and 2019, we selected 36 patients (53 limbs; 83% female) affected by CTS and electrodiagnostic evidence of MUC. In particular, MUC was observed in the right upper limb in 53% of cases and was bilateral in 19 patients (52% of patients; 72% of examined limbs). Though the MUC II was the most common encountered subtype (74%), mixed anastomoses were reported in 55% of explored limbs. No statistical difference was found regarding different MUC subtypes and their combination depending on sex, side (right or left), or laterality (bilateral or monolateral). The detailed distribution of MUC subtypes and the result of NCS in our cohort of 36 patients with CTS and MUC are reported in Table 3 and Table 4, respectively. 

### 3.1. Recordings from ADM Muscle

Among patients with MUC I, the proximal and distal stimulation of the UN yielded a negative response with an average CMAP amplitude significantly increased at the wrist (+21%, 1.68 ± 0.66 mV) compared to below the elbow (*p* = 0.046), whereas the stimulation of MN at the wrist evoked a small positive response (0.5–1 mV) in 16 limbs (30%), with a higher CMAP amplitude at the elbow than wrist stimulation (−86%) (*p* = 0.0001).

### 3.2. Recordings from FDI Muscle

Among patients with MUC II, the proximal (below elbow) and distal (wrist) stimulation of the UN produced a negative response in 92% of patients, with an average of CMAP amplitude significantly increased at the wrist (+80%, 4.14 ± 2.0 mV) compared to below the elbow (*p* = 0. 0001), whereas the stimulation of MN at the wrist evoked a small negative response in 51 examined limbs (96%), with a higher average of CMAP amplitude at the elbow than wrist stimulation (*p* = 0.0001).

### 3.3. Recordings from APB Muscle

Among patients with MUC III, the stimulation of the MN produced a positive onset in 35 arms (66%), while a double component was demonstrated in a further 12 limbs (22%). Though the averages of CMAP amplitude resulting from the stimulation of the MN and UN were not significantly different between the wrist and elbow, the average of CMAP amplitude was slightly reduced at below the elbow compared to the wrist (+24%) after proximal and distal UN stimulation. Moreover, in the presence of MUC III, there was a significant increase in CV upon MN stimulation compared to patients without MUC III (*p* < 0.0001). 

### 3.4. Severity of CTS and Presence of MUC Subtypes 

**Figure 1:**
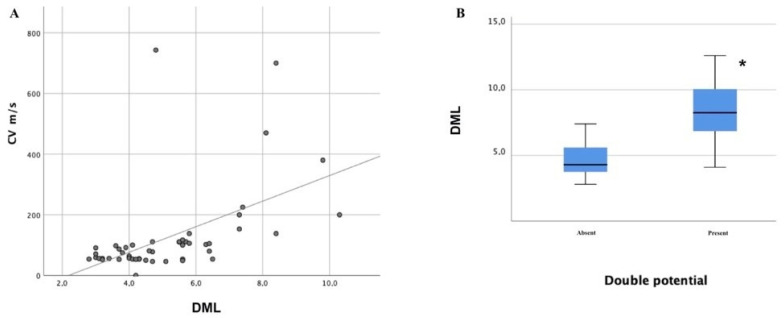
Correlation between DML, double potential, and CV in patients with MUC and CTS. (**A**) The linear correlation between median nerve CV and DML from NCS recording from APB. (**B**) The distribution of double potentials among MUC patients depending on the DML of the median nerve. MUC—median-to-ulnar communicating branch; CTS—carpal tunnel syndrome; CV—conduction velocity; DML—distal motor latency; NCS—nerve conduction studies; APB—abductor brevis pollicis muscle; * *p* < 0.0001.

In our cohort, different severity grades of CTS were found: severe (12 limbs; 23%), moderate (24 limbs; 46%), slight (5 limbs; 9%), minimal (6 limbs; 11%), and normal (6 limbs; 11%). Patients with CTS showed a higher incidence of MUC III (F = 2.87; *p* = 0.034), whereas patients with normal findings on CTS showed a reduced incidence of type III with respect to the ones with severe CTS (*p* = 0.018). Furthermore, the presence of double potential from median stimulation recording from APB appeared more frequently in patients with severe CTS with respect to normal (*p* < 0.0001), minimal (*p* = 0.02), slight (*p* = 0.0001), and moderate (*p* < 0.0001) severity. The CV calculated from MN stimulation recording from APB appeared faster in patients with severe CTS with respect to normal (*p* = 0.004), minimal (*p* = 0.007), slight (*p* = 0.011), and moderate (*p* < 0.0001) severity; the DML obtained from median stimulation recording from APB appeared more prolonged in patients with severe CTS with respect to normal (*p* < 0.0001), minimal (*p* < 0.0001), slight (*p* < 0.0001), and moderate (*p* < 0.0001) severity; the CMAP amplitude at the wrist from median stimulation recording from APB appeared significantly reduced in patients with moderate (*p* = 0.008) and severe (*p* = 0.003) severity with respect to minimal CTS severity; the CMAP amplitude at the elbow from median stimulation recording from APB appeared significantly reduced in patients with moderate (*p* = 0.034) and severe (*p* = 0.008) severity with respect to minimal CTS severity; the DML of the CMAP obtained from UN stimulation recording from FDI appeared significantly prolonged in patients with moderate and severe severity grades compared to minimal (*p* = 0.02; *p* = 0.046) and slight (*p* = 0.16; *p* = 0.038) CTS severity grades. A positive linear correlation between the DML and CV obtained from MN stimulation recording from APB was demonstrated (r = 0.512 and *p* < 0.0001; Figure 1, panel A).

## 4. Discussion

**Figure 2:**
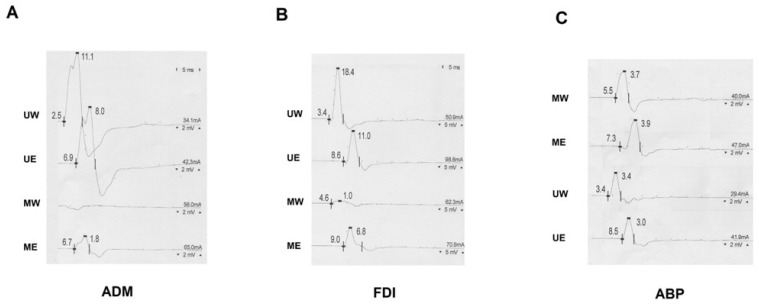
Motor nerve conduction studies from patients with MUC type I (**A**), II (**B**), and III (**C**). (**A**) Recording from ADM, a difference of 3.1 mV was recognizable between U-W and U-BE and an MUC component of 1.8 mV was demonstrated. (**B**) Recording from FDI, a significant drop of 7.4 mV between U-W and U-BE corresponded to a MUC of 6.8 mV, as demonstrated upon ME stimulation. (**C**) A positive onset was evident upon ME but not MW stimulation. MUC—median-to-ulnar communicating branch; ADM—abductor digiti minimi muscle; FDI—first dorsal interosseus muscle; APB—abductor brevis pollicis muscle; U-W—ulnar wrist; U-BE—ulnar below elbow; MW—median wrist; ME—median elbow.

Several studies have explored the prevalence and characteristics of the MUC among healthy subjects [3,4,7,10,13,22,28,29,30], whereas subjects with MN or UN injuries were often excluded. Only a few studies have explored the effects of such communication in a population of patients affected by CTS [5,11,12,21]. Of interest, a study on 63 consecutive patients with bilateral CTS reported signs of MUC in 25% of patients, pointing out that the association between MUC and CTS is not rare [5], and if it occurs, additional electrophysiological changes may appear with the risk of the underestimation of CTS or even false-negative results on NCS [5,11,21]. For instance, patients with CTS and MUC type III present a near normal proximal motor latency of the MN in the presence of prolonged DML, resulting in apparently faster CV [11]. In addition, patients with MUC and CTS might have the partial or complete sparing of the thenar muscles due to the crossover of fibers to the UN [16]. Moreover, MUC type I might mimic an ulnar neuropathy at the elbow in which a reduced-absent response would be expected proximally with the sparing of wrist responses. In this case, the differential diagnosis between ulnar neuropathy at the elbow and MUC type I might be obtained through the stimulation of the MN at the elbow, evoking a wrist response that, when added to the one obtained by stimulation of the UN at the elbow, would equal the response obtained from UN wrist stimulation (Figure 2) [17,26,29]. 

**Figure 3:**
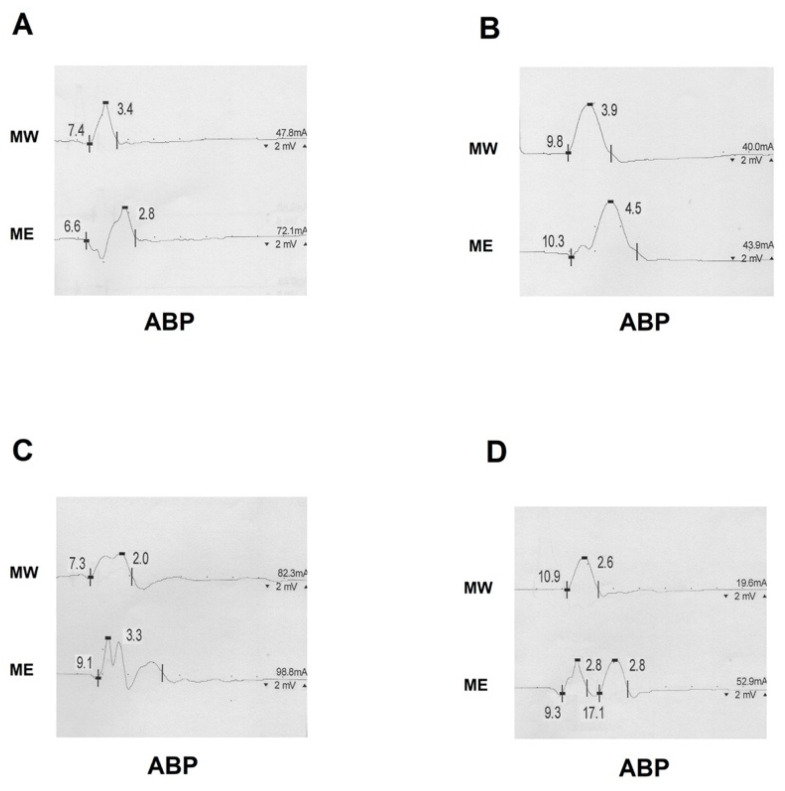
Motor nerve conduction studies from patients with MUC type III and severe CTS. (**A**) A significant positive onset was evident upon ME but not at MW stimulation. (**B**–**D**) A double component was demonstrated upon ME stimulation. (**B**,**C**) The more prolonged the DLM was, the more the two components were spaced apart until they were even separated from the baseline (**D**). MUC—median-to-ulnar communicating branch; APB—abductor brevis pollicis muscle; MW—median wrist; ME—median elbow; DML—distal motor latency.

We confirmed that FDI is the most common MUC-innervated muscle, so is the most useful recording site for the detection of MUC, especially when the FDI atrophy is not severe. A significant increase in the CMAP amplitude at the wrist, compared to the elbow could be easily found while stimulating the UN and recording from FDI and ADM, though not while recording from APB. In our cohort of MUC and CTS patients, there was not a more affected side, according to the existing evidence [5,6,26], although a few studies in healthy subjects have described a slight prevalence of MUC in the right hand [7,10]. Moreover, we reported MUC III in 60% of patients, and several MUC types often coexisted in the same limb (Table 3), in contrast with a majority of the existent studies [10,29]. This aspect is very relevant because many studies on MUC have only considered recordings from APB [11,21,31] or ADM/FDI [18,20,26], and some concern may arise about mistakes in interpreting NCS findings if the possibility of different MUC subtypes is not considered. We found a significant increase in the CMAP amplitude at the wrist compared to below the elbow while stimulating the UN and recording from MUC-innervated muscles. In particular, the best increase in the CMAP amplitude from UN stimulation was detectable from FDI with lower yet significant increases on ADM recordings (Table 4). However, we did not recognize a significant drop in amplitude upon the stimulation of the MN corresponding to the UN gain while recording from APB; indeed, a less prominent drop in amplitude was recorded after the stimulation of the MN (Table 4). This finding was probably due to the fact that the MN neuropathy below the carpal canal may have affected CMAP amplitudes in patients with moderate and severe degrees, making comparisons between MN (affected) and UN (not affected) more difficult. Gutmann et al. attributed this finding to an increased synchronization, as the CMAP tends to have a higher amplitude when the nerve is stimulated distally compared to proximally [5]. Another explanation could be found in the “Riche-Cannieu anastomosis,” a thenar communicating branch of the ulnar nerve [1,32] that usually results in a slightly higher amplitude of the APB-CMAP with UN stimulation at the wrist than the elbow [2,15,23]. Conversely, we found further reliable features in patients with more severe degrees of CTS (Figure 3 and Table 4). 

Previous studies have already described how in the case of moderate and severe CTS degrees, there is a higher CMAP amplitude at the elbow with an initial positive deflection (Figure 2 and Figure 3), which is not seen at the wrist [5,12,21]. The reason for this artefact is that MN axons travelling slower through the carpal canal are overcome by the MUC median-innervated ulnar fibers (not-compressed) being conducting quickly [5,12,21]. In fact, NCS from APB by elbow stimulation record the summation of the normal median response (conducting slower in CTS) and the volume-conducted CMAP recorded from muscles innervated by the anastomotic branch (conducting faster); as a result, the faster volume-conducted response is manifested by an initial positive deflection [16,21]. Additionally, in the presence of a positive onset of the MN CMAP, it might be very difficult to define the real onset of the CMAP and, consequently, the DML, which might be underestimated with a possible underrating of a CTS according to international classifications for severity [33]. This finding has been rarely reported from studies in healthy subjects, where the volume-conducted response has only caused a higher amplitude of the recorded CMAP [21,23]. Hence, the positive deflection alone should not be taken as evidence for MUC. As a further clue for identification, MUC can also cause an apparently fast nerve CV of the MN in the forearm due to the relative sparing of proximal motor latency with respect to the DML [5,8,11,15,21,34]. Of interest, we described a positive correlation between the DML and CV recorded from APB upon MN stimulation (Figure 1, panel A); as a result, the more affected CTS patients tend to have a more prolonged DML and apparently faster CV upon MN studies if measured from the first positive deflection. Furthermore, if the DML is prolonged enough, the cancellation of phase does not occur and a “double CMAP” can be seen in routine studies. Of interest, the frequency of a double component among our patients was increased the more the prolongation of DML was pronounced (Figure 1, panel B). In these more severe CTS, the MUC branch is significant faster than the CMAP of the MN, so the two components are entirely separated with a return to the baseline between each response (Figure 3). This finding cannot be explained by any other physiological or pathological condition [6] and should be considered pathognomonic of MUC III [21]. 

### Limitations

In our study, MUC had an overall lower prevalence than expected (3% of limbs and 4% of patients), and we think that this might have been due to selection bias consistent in patient’s selection among ones with a clinical diagnosis of CTS and previous NCS demonstrating MUC, with an underestimation of the real prevalence of MUC and especially MUC type I and II in real life. Indeed, in our cohort, MUC type III there was more common than other subtypes. Other limitations in patient selection might have been the little attention at the first evaluation paid to the possibility of MUC and the arbitrary limits for the differences in amplitude between the CMAPs at the wrist and elbow stimulation of MN. Furthermore, it has been reported that MUC can alter NCS only if the anastomotic branch contributes more than 5–10% of the total innervations of hand muscles [6]; as a consequence, very thin anastomotic branches are really difficult to detect by electrodiagnostic testing and may have escaped our detection. Moreover, because of we chose the wrist and elbow as stimulation sites for NCS, the very proximal MUC might have escaped detection. We did not consider many factors that may have led to an underestimation of MUC, e.g., height and body weight; indeed, a great representativity of the adipose and muscle tissues might have led to a reduction in CMAP amplitude after proximal stimulation. Finally, a positive wave in median nerve EDX might occur in individuals without MUC as a sign of muscle volume conduction or sensory pre-wave, or it might be generated by the UN co-stimulation of the flexor pollicis brevis. However, in our opinion, these considerations are true in normal NCS and become less relevant in the presence of prominent positive waves in moderate and severe CTS.

## 5. Conclusions

The anastomoses between the MN and UN are usually silent, but they might cause difficulties in interpreting nerve conduction studies, thus leading to misdiagnosis and improper treatment. Indeed, MUC should be always excluded before an ulnar neuropathy at the elbow is confirmed. Additionally, MUC might lead to underestimation in the CTS severity; in these cases, the detection of a positive onset of the CMAP from median nerve stimulation at the elbow, as well as, a paradoxical normal proximal motor latency with prolonged DML and the detection of a double component, might offer reliable clues to identify the presence of MUC in this special population of patients, thus preventing the misinterpretation of electrophysiological data. In particular, the detection of a positive onset or an increased CV can be an important supporting feature in patients with combined CTS and MUC III; in most severe cases of CTS, the presence of a double component is pathognomonic of MUC. Good knowledge of electrodiagnostic features of MUC can ensure an accurate interpretation of NCS in patients affected by entrapment neuropathies, especially in CTS.

## Figures and Tables

**Table 1:** Comparison among different types of median-to-ulnar communicating branch according to MUC classifications.

Type of Communication	Frequency in Healthy Subjects	Distribution	Clinical Suspicion	NCS Findings	Possible Misdiagnosis
MUC type I	2–44% [2,10,13,23,24,25,26,27] Coexisting MUC II in 14–100% of cases [10,23,25,26]	Proximal median to distal ulnar communication innervating the hypothenar muscles.	Absence of hypothenar involvement in the presence of ulnar nerve damage.	Greater CMAP amplitude over ADM recording when stimulating the ulnar nerve at the wrist compared to the elbow.	Ulnar neuropathy at the elbow/cubital tunnel syndrome
MUC type II	8–58% [2,10,13,23,24,25,26,27] Coexisting MUC I in 9–31% of cases [10,23,25,26]	Proximal median to distal ulnar communication innervating the FDI muscle.	Absence of FDI involvement in the presence of ulnar nerve damage.	Greater CMAP amplitude over FDI recording when stimulating the ulnar nerve at the wrist compared to the elbow.	Ulnar neuropathy at the elbow/cubital tunnel syndrome
MUC type III	0.01–30% [2,10,13,24,25,26,27] Coexisting MUC II in 20% of cases [10]	Proximal median to distal ulnar communication innervating the thenar muscles	Absence of thenar involvement in the presence of median nerve damage.	Greater CMAP amplitude over APB recording when stimulating the median nerve at the elbow compared to the wrist.	Carpal tunnel syndrome

MUC—median-to-ulnar communicating branch; NCS—nerve conduction studies; FDI—first dorsal interosseus muscle; ADM—abductor digiti minimi muscle; APB—abductor brevis pollicis muscle.

**Table 2:** Electrodiagnostic criteria for median-to-ulnar communicating branch.

1. Proximal MN evoked CMAP higher at least 2 mV than distal one
2. Initial positive deflection or presence of double component in the MN evoked CMAP at proximal stimulation site recording by ABP
3. MN motor CV over 75 m/s at proximal site of stimulation
4. Distal UN evoked CMAP recorded from ADM or FDI higher at least 2 mV than proximal one
5. Presence of a measurable potential recording from ADM with proximal MN stimulation

MN—median nerve; UN—ulnar nerve; CMAP—compound motor action potential; CV—conduction velocity; ADM—abductor digit minimi muscle; FDI—first dorsal interosseus muscle.

**Table 3:** Features of median-to-ulnar communicating branch in 36 patients with carpal tunnel syndrome and MUC. MUC—median-to-ulnar communicating branch.

	MUC I	MUC II	MUC III	Total (Limbs)
Limbs (*n*, %)	18 (34%)	39 (74%)	32 (60%)	53
Sex (males, %)	2 (11%)	6 (15%)	4 (13%)	9 (17%)
Side (right, %)	8 (44%)	19 (49%)	20 (63%)	28 (53%)
Isolated communication (*n*, %)	0	10 (26%)	11 (34%)	21 (40%)
Coexistent MUC I (*n*, %)	/	18 (46%)	9 (28%)	/
Coexistent MUC II (*n*, %)	18 (100%)	/	20 (63%)	/
Coexistent MUC III (*n*, %)	9 (50%)	20 (51%)	/	/

**Table 4:** Nerve conduction studies in patients with carpal tunnel syndrome and median-to-ulnar communicating branch. Values are expressed as means with standard deviations or percentages.

Recording Site	MUC I	MUC II	MUC III	Total Limbs
*ADM ulnar nerve*				
DML (ms)	2.8 ± 0.6	2.8 ± 0.5	2.7 ± 0.5	2.7 ± 0.5
CMAP-AW (mV)	10.5 ± 2.3	10.5 ± 2.4	10.8 ± 2.0	10.6 ± 2.3
CMAP-AE (mV)	8.9 ± 2.4	9.1 ± 2.4	9.2 ± 2.2	9.1 ± 2.3
CV (m/s)	58.0 ± 7.6	57.8 ± 4.9	59.5 ± 4.7	58.7 ± 6.9
Positive onset (*n*, %)	2 (11%)	2 (5%)	1 (3%)	4 (8%)
Double component (*n*, %)	0	0	0	0
Ulnar Gain in amplitude mV (%)	1.7 ± 0.7 (21%)	1.2 ± 0.9 (19%)	1.4 ± 0.8 (22%)	1.2 ± 0.9 (19%)
*ADM median nerve*				
DML (ms)	5.6 ± 2.0	5.1 ± 1.9	5.2 ± 2.2	5.1 ± 1.9
CMAP-AW (mV)	0.2 ± 0.6	0.3 ± 0.6	0.4 ± 0.7	0.3 ± 0.6
CMAP-AE (mV)	1.1 ± 0.7	0.9 ± 0.7	0.7 ± 0.7	0.8 ± 0.7
Positive onset (*n*, %)	6 (33%)	6 (15%)	3 (9%)	15 (28%)
Double component (*n*, %)	0	0	0	0
Median drop in amplitude mV (%)	0.9 ± 0.4 (86%)	0.5 ± 0.5 (73%)	0.3 ± 0.4 (63%)	0.4 ± 0.5 (73%)
*FDI ulnar nerve*				
DML (ms)	3.4 ± 0.3	3.5 ± 0.4	3.6 ± 0.5	3.5 ± 0.4
CMAP-AW (mV)	11.9 ± 4.7	11.2 ± 4.4	10.4 ± 3.8	11.3 ± 4.4
CMAP-AE (mV)	7.1 ± 4.0	7.0 ± 3.7	6.8 ± 3.3	7.3 ± 3.9
CV (m/s)	55.8 ± 5.8	54.3 ± 7.1	52.6 ± 6.6	54.6 ± 6.9
Positive onset (*n*, %)	2 (11%)	3 (8%)	0	4 (8%)
Double component (*n*, %)	0	0	0	0
Ulnar Gain in amplitude mV (%)	4.9 ± 2.4 (93%)	4.1 ± 2.0 (80%)	3.4 ± 1.5 (67%)	3.9 ± 1.1 (73%)
*FDI median nerve*				
DML (ms)	4.7 ± 0.8	4.9 ± 1.1	5.3 ± 1.5	4.8 ± 1.1
CMAP-AW (mV)	0.6 ± 4.0	0.7 ± 0.6	0.8 ± 1.2	0.8 ± 0.9
CMAP-AE (mV)	4.0 ± 2.6	3.6 ± 2.2	3.5 ± 2.0	3.5 ± 2.2
Positive onset (*n*, %)	0	0	0	0
Double component (*n*, %)	0	0	0	0
Median drop in amplitude mV (%)	3.3 ± 2.0 (81%)	2.9 ± 1.7 (79%)	2.6 ± 1.4 (73%)	2.7 ± 1.6 (74%)
*APB median nerve*				
DML (ms)	5.3 ± 2.6	5.2 ± 2.2	6.2 ± 2.4	5.3 ± 2.2
CMAP-AW (mV)	7.1 ± 3.1	7.0 ± 3.5	5.0 ± 3.4	6.3 ± 3.7
CMAP-AE (mV)	7.1 ± 3.0	7.4 ± 3.6	5.4 ± 3.4	6.6 ± 4.0
CV (m/s)	99.9 ± 58.7	103.1 ± 118.7	172.8 ± 113.8	123 ± 145
Positive onset (*n*, %)	12 (67%)	22 (56%)	28 (88%)	35 (66%)
Double component (*n*, %)	3 (17%)	5 (13%)	11 (34%)	12 (23%)
Median drop in amplitude mV (%)	0.1 ± 0.5 (1%)	0.4 ± 0.7 (5%)	0.5 ± 0.7 (6%)	0.5 ± 0.7 (5%)
*APB ulnar nerve*				
DML (ms)	3.1 ± 0.8	3.3 ± 0.8	3.2 ± 0.7	3.2 ± 0.7
CMAP-AW (mV)	4.2 ± 1.5	4.5 ± 2.0	4.3 ± 1.8	4.6 ± 2.2
CMAP-AE (mV)	3.6 ± 1.4	3.7 ± 1.5	3.6 ± 1.4	3.8 ± 1.6
Positive onset (*n*, %)	3 (17%)	9 (23%)	9 (28%)	11 (21%)
Double component (*n*, %)	0	0	0	0
Ulnar Gain in amplitude mV (%)	0.5 ± 0.6 (19%)	0.7 ± 0.7 (21%)	0.8 ± 0.6 (24%)	0.7 ± 0.7 (21%)

MUC—median-to-ulnar communicating branch; ADM—abductor digiti minimi muscle; FDI—first dorsal interosseus muscle; APB—abductor brevis pollicis muscle; CMAP-AW/E—compound motor action potential at the wrist/elbow; ADM ulnar nerve—ulnar nerve stimulation while recording from ADM muscle; ADM median nerve—median nerve stimulation while recording from ADM muscle; FDI ulnar nerve—ulnar nerve stimulation while recording from FDI muscle; FDI median nerve—median nerve stimulation while recording from FDI muscle; APB median nerve—median nerve stimulation while recording from APB muscle; APB ulnar nerve—ulnar nerve stimulation while recording from APB muscle; DML—distal motor latency; CV—conduction velocity.

## Data Availability

The data that support the findings of this study are available from the corresponding author upon reasonable request.
